# Effects of Velocity-Based Resistance Training on Renal Function and Metabolic Health in Kidney Transplant Recipients: Protocol for a Pilot Randomized Controlled Trial

**DOI:** 10.2196/94010

**Published:** 2026-07-10

**Authors:** Jhonatan Camilo Peña Ibagon, Fermina Vasquez Osorio, Carlos Andrés Collazos Morales, Blanca Elpidia Tovar, William Felipe Martin1, Yordan Rene Pardo, Luis Andres Tellez

**Affiliations:** 1 Fundación Universitaria del Área Andina Bogota, Bogota D.C. Colombia; 2 Universidad del Sinú Cordoba, Córdoba Colombia; 3 Universidad Manuela Beltrán Bogota, Bogota D.C. Colombia; 4 Corporación Unificada Nacional de Educación Superior Bogota, Bogota D.C. Colombia

**Keywords:** kidney transplantation, resistance training, velocity-based training, pilot randomized controlled trial, renal function, metabolic health, exercise intervention, creatinine, HDL cholesterol

## Abstract

**Background:**

Kidney transplant recipients present reduced physical function and a high prevalence of cardiometabolic complications, which increase cardiovascular risk and compromise long-term graft outcomes. Resistance training has demonstrated beneficial effects in this population; however, previous interventions have shown heterogeneity in load prescription and have not incorporated objective monitoring of movement velocity. Velocity-based resistance training (VBT) allows precise regulation of exercise intensity and fatigue, potentially improving the safety and individualization of exercise prescription in clinical populations.

**Objective:**

This study aims to evaluate the effects of a 12-week VBT program on renal function and metabolic health in kidney transplant recipients and to compare 2 different load-control strategies based on movement velocity.

**Methods:**

This pilot randomized controlled trial will include adult kidney transplant recipients with stable graft function. Participants will be randomly assigned (1:1) to either a maximal velocity group, in which sets will be terminated at a 20% velocity loss threshold, or a constant submaximal velocity group, in which participants will perform repetitions at 50% of the participant’s individual maximal velocity. Both groups will complete 3 supervised training sessions per week for 12 weeks with real-time velocity monitoring. Primary outcomes will include renal and metabolic health domains assessed through venous blood analysis. Serum creatinine will be the prespecified hierarchical primary renal end point, and high-density lipoprotein cholesterol will be the prespecified hierarchical primary metabolic end point. Estimated glomerular filtration rate will be calculated using the Chronic Kidney Disease Epidemiology Collaboration equation. Secondary outcomes will include blood pressure, body composition, muscular strength, metabolic syndrome criteria, and the force-velocity profile. Data will be analyzed using analysis of covariance and linear mixed-effects models following a predefined hierarchical inferential strategy.

**Results:**

The study was initiated in September 2025. Participant recruitment and the intervention phase have been completed. All 14 participants completed the 12-week training program, and no participants were lost to follow-up. Preintervention and postintervention data collection has been completed according to the study protocol. The study database has been cleaned and locked, and statistical analyses are currently underway. Publication of the primary study results is anticipated in late 2026.

**Conclusions:**

This study introduces the implementation of VBT in kidney transplant recipients. The findings are expected to provide evidence on the feasibility and potential benefits of this approach and may support the integration of exercise professionals into multidisciplinary transplant care teams.

**Trial Registration:**

ClinicalTrials.gov NCT07370727; https://clinicaltrials.gov/study/NCT07370727

**International Registered Report Identifier (IRRID):**

DERR1-10.2196/94010

## Introduction

Kidney transplantation is the treatment of choice for patients with end-stage chronic kidney disease, as it significantly improves survival and quality of life compared with dialysis [1,2]. However, despite these benefits, kidney transplant recipients continue to present a high risk of metabolic and cardiovascular complications that negatively affect long-term graft and patient survival [3,4]. These complications are closely associated with modifiable factors, such as physical inactivity, increased adiposity, insulin resistance, and the presence of metabolic syndrome, the prevalence of which is considerably elevated in this population [5-7]. Metabolic syndrome has been associated with an increased risk of graft dysfunction, cardiovascular events, and mortality, highlighting the importance of implementing interventions aimed at improving metabolic and functional health in kidney transplant recipients [8,9].

In this context, physical exercise has been recognized as a safe and effective intervention for improving physical fitness, body composition, quality of life, and several cardiometabolic markers in kidney transplant recipients [10-13]. Evidence derived from randomized controlled trials and systematic reviews has demonstrated that structured exercise programs can improve muscular strength, cardiorespiratory capacity, and certain cardiovascular risk factors without compromising graft function when appropriately supervised [10,11,14-16]. In particular, resistance training has shown positive effects on muscle mass, insulin sensitivity, and metabolic health, which are key factors in the clinical prognosis of this population [12,14].

Nevertheless, despite these benefits, the results of intervention studies have been heterogeneous and inconsistent, particularly with respect to metabolic and renal outcomes. One of the main limitations explaining this inconsistency is the lack of standardization in the programming and periodization of resistance training load components. Recently, a scoping review that characterized resistance training programs in kidney transplant recipients identified substantial heterogeneity in critical variables, such as intensity, volume, number of sets, repetitions, and recovery intervals, with a predominance of prescribing intensity based on a percentage of 1-repetition maximum as the primary method [17]. This methodological heterogeneity limits the reproducibility of interventions and hinders the identification of optimal resistance training prescription models for this clinical population.

Importantly, this review also reported that movement velocity, a key variable for the precise control of intensity and neuromuscular fatigue, has not been considered a central component of resistance training programming for kidney transplant recipients [17]. Movement velocity is one of the most valid and sensitive indicators for load prescription in resistance training, allowing the estimation of relative intensity from the first repetition, the control of fatigue through monitoring of velocity loss, and the optimization of neuromuscular and metabolic adaptations [18-21]. The absence of this variable in previously studied resistance training programs represents a critical limitation, particularly in clinical populations in which individualization and precise stimulus control are essential.

Over the last decade, velocity-based resistance training (VBT) has emerged as an innovative paradigm that enables individualized, objective, and precise prescription of training load [18-21]. This approach allows real-time adjustment of intensity, control of effective training volume, and optimization of training safety and efficacy, characteristics that are particularly relevant in clinical populations such as kidney transplant recipients.

However, to date, no randomized controlled trials have evaluated the effects of a resistance training program prescribed and monitored using movement velocity on renal function and metabolic health in kidney transplant recipients. This lack of evidence represents a critical gap in the scientific literature, especially considering the previously documented heterogeneity in the periodization of load components and the absence of objective intensity control in this population [17].

Although serum creatinine is widely used as a clinical marker of renal function in kidney transplant recipients, its interpretation in the context of resistance training requires caution. Increases in skeletal muscle mass induced by training may lead to elevations in serum creatinine independent of changes in renal function. Despite this limitation, creatinine remains a clinically relevant and routinely monitored biomarker in this population. Therefore, its inclusion as a primary renal outcome is justified, but results will be interpreted alongside complementary indicators and within the context of potential training-induced muscular adaptations. In addition, high-density lipoprotein cholesterol (HDL-C) was selected as the primary metabolic outcome because of its sensitivity to exercise interventions and its established role as a protective cardiovascular factor in transplant recipients.

Therefore, this study aims to describe the protocol for a pilot randomized controlled trial designed to evaluate the preliminary effects of a VBT program on renal function and metabolic health in kidney transplant recipients. In addition to assessing preliminary efficacy, this pilot trial aims to evaluate feasibility indicators, including the recruitment rate, intervention adherence, and safety profile. The findings of this study are expected to provide foundational evidence to inform the design of future adequately powered randomized controlled trials in this clinical population.

## Methods

### Study Design

This study is designed as a pilot randomized, parallel-group controlled clinical trial with a 1:1 allocation ratio to assess the feasibility, safety, and preliminary efficacy of 2 VBT strategies in kidney transplant recipients. The pilot nature of the trial aims to generate preliminary estimates of effect size and variability to inform future adequately powered randomized controlled trials in this population. The protocol has been developed in accordance with the SPIRIT (Standard Protocol Items: Recommendations for Interventional Trials) guidelines to ensure methodological rigor, transparency, and reproducibility [22]. A completed SPIRIT 2025 checklist is provided as Multimedia Appendix 1.

### Feasibility Outcome

Feasibility outcomes will include recruitment rate, intervention adherence, retention rate, safety, and data completeness. Recruitment will be considered successful if at least 2 participants per month are enrolled, considering the clinical characteristics and limited availability of the target population. Adherence will be defined as attendance at ≥80% of the scheduled sessions. Retention will be considered acceptable if ≥85% of participants complete the intervention. Safety will be assessed through monitoring of adverse events, with an acceptable threshold defined as <10% of participants experiencing exercise-related complications. Data completeness will be considered adequate if ≥90% of the expected data are collected.

### Ethical Considerations

This study was approved by the research ethics committee of the Fundación Universitaria del Área Andina (approval no. 15; dated April 22, 2025) and will be conducted in accordance with the ethical standards of the institutional research committee and the principles of the Declaration of Helsinki. Furthermore, the trial protocol was prospectively registered at ClinicalTrials.gov (National Library of Medicine) under the identifier NCT07370727, ensuring transparency, traceability, and public access to study information.

All participants will be fully informed about the study procedures, potential risks, and benefits, and will provide written informed consent prior to enrollment. Participation will be voluntary, and participants may withdraw from the study at any time without consequence. Participant confidentiality will be ensured by assigning anonymized identification codes and storing data in secure, password-protected databases accessible only to the research team. No personal identifiers will be disclosed in any publication derived from this study. Participants will not receive financial compensation but will benefit from supervised exercise sessions and health monitoring throughout the intervention.

### Participants and Eligibility Criteria

Men and women aged 18 to 50 years with a history of kidney transplantation and who are at least 12 months after transplantation will be invited to participate, provided they have stable graft function during the previous 6 months. Stable graft function will be defined as resting serum creatinine levels below 1.5 mg/100 mL, an estimated glomerular filtration rate of ≥60 mL/min/1.73 m², no variation greater than +10% to –10% in renal function over the previous 3 months, the absence of acute rejection episodes in the last 6 months, no recent changes in immunosuppressive therapy, and proteinuria levels below 300 mg/g. All participants will be required to have written medical clearance from their treating nephrologist authorizing participation in moderate to vigorous physical exercise and to sign informed consent prior to inclusion in the study. Individuals with active autoimmune disorders, a recent history (≤6 months) of coronary artery disease, osteomuscular limitations that could prevent safe participation in the intervention, a diagnosis of diabetes mellitus before or after transplantation, active infection, or use of immunosuppressive agents requiring exercise restriction, such as mechanistic target of rapamycin inhibitors, at the time of enrollment will be excluded. Recruitment will be conducted through a public call distributed by the *Asociación Colombiana de Deportistas Trasplantados*, followed by an initial telephone screening. Potentially eligible participants will subsequently be invited to an in-person evaluation, during which clinical history will be obtained, eligibility criteria will be verified, and informed consent will be obtained.

### Sample Size

The sample size was estimated based on the primary outcome domains of the study, corresponding to renal function and metabolic health, specifically changes in serum creatinine and HDL-C. The calculation was informed by evidence from previous supervised exercise interventions in kidney transplant recipients that reported clinically meaningful changes in these physiological parameters [10,16,23]. Considering the exploratory nature of this pilot trial, the sample size was not primarily determined to test hypotheses or detect statistically significant between-group differences. Rather, a pragmatic approach was adopted to include 14 participants (7 participants per group), which was considered sufficient to evaluate key feasibility outcomes, such as recruitment, adherence, retention, and safety, as well as to provide preliminary estimates of variability and effect size. These estimates are intended to inform the design and sample size calculation of a future fully powered randomized controlled trial. Therefore, any between-group comparisons conducted in this study will be considered exploratory and interpreted with caution. The calculation was performed using the Power and Sample Size Calculation software (Vanderbilt University).

### Randomization and Allocation Concealment

Participants will be assigned to intervention groups using a block randomization procedure with a 1:1 allocation ratio, generated through a computerized random sequence generator. To ensure allocation concealment, a centralized online randomization platform (Research Randomizer, Social Psychology Network) will be used, to which only the methodological coordinator will have access. This coordinator will not be involved in outcome assessment or data analysis. After eligibility criteria are confirmed, informed consent is obtained, and baseline assessments are completed. Participant information will be entered into the platform, which automatically assigns participants to 1 of the 2 intervention groups using an encrypted system, ensuring allocation unpredictability and minimizing selection bias.

### Blinding

Due to the nature of the intervention, blinding of participants and trainers will not be possible. However, a single-blind design will be implemented for outcome assessors and data analysts. Evaluators responsible for biochemical, anthropometric, and physiological measurements will remain blinded to group allocation, and statistical analysis will be conducted using a coded dataset in which groups will be identified as group A and group B. This procedure will minimize measurement and analysis bias, strengthening the internal validity of the study.

### Outcome Measures

Renal function markers will comprise serum creatinine, urea, blood urea nitrogen, and uric acid, which are biomarkers widely used in the clinical follow-up of kidney transplant recipients [24,25]. Estimated glomerular filtration rate will be calculated using the Chronic Kidney Disease Epidemiology Collaboration equation based on serum creatinine values, age, and sex. For inferential analysis, serum creatinine will be the prespecified hierarchical primary renal end point. It is acknowledged that resistance training may increase skeletal muscle mass, which could influence serum creatinine levels independently of renal function.

Metabolic health will be assessed through fasting glucose, total cholesterol, HDL-C, low-density lipoprotein cholesterol, and triglycerides. HDL-C will be the prespecified primary metabolic marker because of its sensitivity to exercise-induced adaptations and its clinical relevance in cardiovascular risk reduction.

Secondary outcomes will include clinical, anthropometric, body composition, physical fitness, and neuromuscular function variables. A predefined analytic hierarchy will be established, prioritizing renal function (serum creatinine) as the primary outcome, followed by metabolic health indicators (HDL-C) and secondary outcomes related to physical performance and cardiovascular risk.

### Instruments and Procedures

Assessments will be performed at 2 time points, baseline (preintervention assessment) and after completion of the 12-week intervention (postintervention assessment), by trained evaluators blinded to group allocation, following standardized protocols to ensure the validity, reliability, and reproducibility of the measurements.

Primary outcomes will be evaluated through venous blood analysis processed at a certified clinical laboratory (Instituto de Diagnóstico Médico). Samples will be collected between 6 AM and 9 AM by specialized nursing staff under standardized conditions, including a minimum 12-hour fasting period and abstention from physical exercise for 24 hours prior to sampling [26].

Blood pressure will be measured using a validated digital sphygmomanometer, following standardized protocols with participants seated and at rest for at least 5 minutes [27]. Waist and hip circumferences will be assessed using a nonelastic anthropometric tape according to International Society for the Advancement of Kinanthropometry protocols and will be used along with biochemical markers to diagnose metabolic syndrome according to the National Cholesterol Education Program Adult Treatment Panel III criteria [27,28]. All anthropometric measurements will be conducted by evaluators certified under International Society for the Advancement of Kinanthropometry standards, and both intrarater and interrater technical errors of measurement will be calculated to ensure data quality and reliability. Additionally, a metabolic risk index will be calculated by transforming metabolic variables into standardized scores using previously validated methodologies [29].

Body composition will be assessed using segmental bioelectrical impedance analysis (Tanita Corp), allowing estimation of body mass, muscle mass, and body fat percentage [30]. Height will be measured using a digital stadiometer following standardized anthropometric protocols.

Muscle strength will be assessed using handgrip dynamometry with a validated digital dynamometer (Takei Scientific Instruments Co Ltd), recording the highest value obtained from maximal attempts [31]. Functional strength will also be evaluated using push-up and horizontal jump tests, which are used as indicators of global neuromuscular performance [32]. A general strength index will be calculated by integrating standardized scores (*Z* scores) from these tests.

The force-velocity profile will be assessed using a validated linear encoder (T-Force System; Ergotech), a device widely used to quantify mean propulsive velocity and estimate neuromuscular profiles during resistance exercises [33]. The assessment will be conducted using a Smith machine and will include bench press, shoulder press, and half squat exercises. The eccentric phase will be performed at a controlled velocity (approximately 0.50-0.65 m/s), and the concentric phase will be performed at maximal intended velocity. The load will be progressively increased until the mean propulsive velocity is <0.8 m/s. This procedure has demonstrated high validity and reliability [18,33].

Physical activity level will be assessed using the International Physical Activity Questionnaire, and perceived physical fitness will be assessed using the International Fitness Scale, both validated in Colombian populations [34,35].

### Intervention: Training Protocols

The VBT program will be conducted over 12 weeks, with 3 weekly sessions on nonconsecutive days, totaling 36 sessions. Each session will last approximately 50 to 80 minutes and will include warm-up, main training, and cool-down phases. Exercises will include bench press, half squat, and military press, performed on a Smith machine, with velocity monitored using a linear encoder.

Participants will be randomly assigned to 1 of 2 intervention groups. Group A—maximal velocity with velocity loss control: participants will perform the concentric phase at maximal voluntary velocity and the eccentric phase in a controlled manner. Sets will be terminated when mean propulsive velocity decreases by 20% relative to the fastest repetition in the set. The load will be progressively increased following the principle of progressive overload. The detailed load progression and training parameters for the maximal velocity group are presented in Table 1.

**Table 1:** Load progression and training parameters for the maximal velocity group.

Sessions	Intensity (% 1-repetition maximum)	Sets per exercise	Termination criterion (% velocity loss)	Rest between sets (minutes)	Rest between exercises (minutes)
1-6	20	2	20	1	3
7-12	30	2	20	1	3
13-18	40	3	20	2	3
19-24	50	3	20	2	3
25-36	60	4	20	2	3

Group B—constant submaximal velocity training: participants in this group will perform the same exercises, with identical load progression, number of sets, and recovery periods. However, repetitions will be performed at a concentric velocity equivalent to 50% of the previously determined individual maximal velocity, eliminating the relative velocity loss criterion as the indicator for set termination. In this group, training volume will be standardized using a fixed number of repetitions per set. The detailed load progression and training parameters for the maximal velocity group are presented in Table 2.

**Table 2:** Load progression and training parameters for the constant submaximal velocity group.

Sessions	Intensity (% 1-repetition maximum)	Repetitions per set	Sets per exercise	Rest between sets (minutes)	Rest between exercises (minutes)
1-6	20	30	2	1	3
7-12	30	25	2	1	3
13-18	40	20	3	2	3
19-24	50	18	3	2	3
25-36	60	15	4	2	3

All training sessions will be supervised by a professional in sports sciences with experience in resistance training and clinical rehabilitation, who will ensure proper exercise execution and compliance with the established protocol. During each session, mean propulsive velocity, external load, number of repetitions, number of sets, and program adherence will be recorded using a linear encoder and a digital monitoring system. This approach will ensure proper implementation of the intervention, optimize individualized load prescription, and guarantee participant safety.

At the end of each session, a cool-down period of approximately 10 minutes will be performed, including static stretching exercises targeting the main muscle groups involved, such as the pectorals, deltoids, quadriceps, hamstrings, and gluteal muscles, to facilitate neuromuscular recovery and reduce injury risk. Adherence to the program will be defined as attendance ≥85% of the scheduled sessions, and any adverse events will be documented and evaluated by the research team. The protocol has been designed according to international recommendations for resistance training in clinical populations, ensuring appropriate conditions of safety, progression, and individualization of training [7,16,19].

### Safety Monitoring

Adverse events will be continuously monitored throughout the intervention by the research team. Any exercise-related complication or abnormal clinical response will be documented and evaluated. Given the low-risk nature of the intervention and the pilot design of the study, a formal data monitoring committee has not been established. However, safety oversight will be conducted by the principal investigator and the clinical collaborators. Criteria for discontinuation will include any medical condition that contraindicates continued participation in the intervention.

### Data Management

All study data will be recorded in a secure digital database with restricted access. Data entry will be verified through double-check procedures, and periodic quality-control checks will be performed to ensure accuracy, consistency, and completeness of the dataset. Participant information will be coded to preserve confidentiality throughout data management and analysis.

### Statistical Analysis

Analyses will be conducted according to the intention-to-treat principle, including all participants according to their assigned group. Missing data will be handled using appropriate imputation methods, and sensitivity analyses will be conducted to assess the robustness of the results. Continuous variables will be expressed as mean and SD (or median and IQR when normality assumptions are not met), and categorical variables will be expressed as frequencies and percentages. Normality of the data will be assessed using the Shapiro-Wilk test and graphical inspection of residuals. A statistical significance level of *P*<.05 will be considered, and 95% CI will be reported. The effect of the intervention will be primarily analyzed using an analysis of covariance model, using the postintervention value as the dependent variable, group as a fixed factor, and baseline value as a covariate, to adjust for potential baseline differences and improve estimator precision. As a complementary analysis, and to appropriately account for the longitudinal structure of the data, linear mixed-effects models will be used with fixed effects for group, time (before and after), and the group×time interaction, including a random intercept for each participant. Given that the study will include 2 primary outcomes (renal function and metabolic health), a predefined analytic hierarchy will be applied, with renal function evaluated first using serum creatinine, followed by metabolic health markers, particularly HDL-C, to control the type I error rate. Categorical variables, such as the presence of metabolic syndrome, will be compared between groups using the Fisher exact test. Effect size will be estimated using Cohen *d*, and its magnitude will be interpreted as trivial (<0.2), small (0.2-0.59), moderate (0.60-1.19), large (1.2-2.0), or very large (>2.0), according to established criteria [36]. All statistical analyses will be performed using SPSS (version 23; IBM Corp).

## Results

The study was approved by the Research Ethics Committee of Fundación Universitaria del Área Andina (approval 15, dated April 22, 2025) and prospectively registered at ClinicalTrials.gov (NCT07370727). The study was initiated in September 2025. Participant recruitment and the intervention phase have been completed. All 14 participants completed the 12-week training program, and no participants were lost to follow-up. Preintervention and postintervention data collection were completed according to the study protocol. The study database has been cleaned and locked, and statistical analyses are currently underway. Publication of the primary study results is anticipated in late 2026.

## Discussion

This study is designed to evaluate the feasibility, safety, and preliminary effects of a VBT program in kidney transplant recipients. It is anticipated that the implementation of VBT will allow more individualized and precise control of training load, which may contribute to improvements in renal function markers and metabolic health indicators, particularly serum creatinine and HDL-C. Additionally, the study is expected to provide valuable information regarding the adherence, tolerability, and safety of this training approach in a clinical population that has historically been underrepresented in exercise intervention studies.

Previous studies examining resistance training in kidney transplant recipients have demonstrated beneficial effects on physical function, quality of life, and certain metabolic parameters [10]. However, most of these interventions have been based on traditional load-prescription methods using percentages of 1-repetition maximum, with limited reporting of key training variables, such as movement velocity, volume distribution, and fatigue management. In contrast, this study introduces VBT as a novel methodological approach in this population. By incorporating mean propulsive velocity as a real-time monitoring variable, this protocol aims to enhance the individualization of training stimuli and provide a more objective and responsive method for load adjustment. This represents a potential advancement over conventional training paradigms, particularly in clinical populations in which safety and precise dose control are critical.

This study has several strengths. First, it adopts a randomized controlled design, which enhances internal validity. Second, it incorporates an objective and innovative method for training-load prescription through velocity-based monitoring. Third, it targets a specific clinical population for which evidence regarding optimized resistance training strategies is limited. However, several limitations must be acknowledged. As a pilot randomized controlled trial, the sample size is small and not intended to provide definitive evidence of effectiveness. Therefore, findings related to between-group differences should be interpreted with caution. Additionally, the inability to blind participants and trainers may introduce performance bias, although efforts have been made to minimize this through blinded outcome assessment and data analysis. Finally, the single-center nature of the study may limit the generalizability of the findings.

The findings of this pilot study are expected to inform the design of a future fully powered randomized controlled trial. Future research should consider larger sample sizes, multicenter designs, and longer follow-up periods to evaluate the long-term effects of VBT on renal function, metabolic health, and clinical outcomes in kidney transplant recipients. Additionally, further studies may explore the integration of VBT with other lifestyle interventions, such as nutritional strategies, to optimize patient outcomes.

The results of this study will be disseminated through publication in peer-reviewed scientific journals and presentations at national and international conferences in the fields of sports science, rehabilitation, and clinical exercise physiology. Additionally, findings will be shared with health care professionals and relevant institutions involved in the care of kidney transplant recipients, with the aim of contributing to evidence-based exercise prescription in this population.

## Appendix

Multimedia Appendix 1 SPIRIT 2025 checklist.

## Abbreviations

HDL-C: high-density lipoprotein cholesterol
SPIRIT: Standard Protocol Items: Recommendations for Interventional Trials
VBT: velocity-based resistance training

## Data Availability

Deidentified participant data will be available from the corresponding author upon reasonable request following publication of the primary results.

## References

[1] Wolfe RA, Ashby VB, Milford EL, Ojo AO, Ettenger RE, Agodoa LY, Held PJ, Port FK (1999). Comparison of mortality in all patients on dialysis, patients on dialysis awaiting transplantation, and recipients of a first cadaveric transplant. N Engl J Med.

[2] Hariharan S, Israni AK, Danovitch G (2021). Long-term survival after kidney transplantation. N Engl J Med.

[3] Cohen E, Korah M, Callender G, Belfort de Aguiar R, Haakinson D (2020). Metabolic disorders with kidney transplant. Clin J Am Soc Nephrol.

[4] Goldsmith D, Pietrangeli CE (2010). The metabolic syndrome following kidney transplantation. Kidney Int Suppl.

[5] Dedinská I, Palkoci B, Miklušica J, Osinová D, Galajda P, Mokáň M (2017). Metabolic syndrome and new onset diabetes after kidney transplantation. Diabetes Metab Syndr.

[6] Saklayen MG (2018). The global epidemic of the metabolic syndrome. Curr Hypertens Rep.

[7] Ojo AO (2006). Cardiovascular complications after renal transplantation and their prevention. Transplantation.

[8] Salari M, Yaghoubi MA, Miri M, Mehrad-Majd H, Hami M (2023). Association of metabolic syndrome and hyperuricemia in the recipients of kidney transplants: a single-center study. Iran J Kidney Dis.

[9] Sharif A, Chakkera H, de Vries AP, Eller K, Guthoff M, Haller MC, Hornum M, Nordheim E, Kautzky-Willer A, Krebs M, Kukla A, Kurnikowski A, Schwaiger E, Montero N, Pascual J, Jenssen TG, Porrini E, Hecking M (2024). International consensus on post-transplantation diabetes mellitus. Nephrol Dial Transplant.

[10] Wilkinson TJ, Bishop NC, Billany RE, Lightfoot CJ, Castle EM, Smith AC, Greenwood SA (2021). The effect of exercise training interventions in adult kidney transplant recipients: a systematic review and meta-analysis of randomised control trials. Phys Ther Rev.

[11] Janaudis-Ferreira T, Tansey CM, Mathur S, Blydt-Hansen T, Lamoureaux J, Räkel A, de Sousa Maia NP, Bussières A, Ahmed S, Boruff J (2021). The effects of exercise training in adult solid organ transplant recipients: a systematic review and meta-analysis. Transpl Int.

[12] Takahashi A, Hu SL, Bostom A (2018). Physical activity in kidney transplant recipients: a review. Am J Kidney Dis.

[13] De Smet S, Van Craenenbroeck AH (2021). Exercise training in patients after kidney transplantation. Clin Kidney J.

[14] Hernández Sánchez S, Carrero JJ, Morales JS, Ruiz JR (2021). Effects of a resistance training program in kidney transplant recipients: a randomized controlled trial. Scand J Med Sci Sports.

[15] Lima PS, de Campos AS, de Faria Neto O, Ferreira TC, Amorim CE, Stone WJ, Prestes J, Garcia AM, Urtado CB (2021). Effects of combined resistance plus aerobic training on body composition, muscle strength, aerobic capacity, and renal function in kidney transplantation subjects. J Strength Cond Res.

[16] Roi GS, Mosconi G, Totti V, Angelini ML, Brugin E, Sarto P, Merlo L, Sgarzi S, Stancari M, Todeschini P, La Manna G, Ermolao A, Tripi F, Andreoli L, Sella G, Anedda A, Stefani L, Galanti G, Di Michele R, Merni F, Trerotola M, Storani D, Nanni Costa A (2018). Renal function and physical fitness after 12-mo supervised training in kidney transplant recipients. World J Transplant.

[17] Peña JC, Sánchez-Guette L, Lombo C, Pinto E, Collazos C, Tovar B, Bonilla DA, Cardozo LA, Tellez LA (2025). Characterization of load components in resistance training programs for kidney transplant recipients: a scoping review. Sports (Basel).

[18] González-Badillo JJ, Sánchez-Medina L (2010). Movement velocity as a measure of loading intensity in resistance training. Int J Sports Med.

[19] Pareja-Blanco F, Rodríguez-Rosell D, Sánchez-Medina L, Gorostiaga EM, González-Badillo JJ (2014). Effect of movement velocity during resistance training on neuromuscular performance. Int J Sports Med.

[20] Sánchez-Medina L, González-Badillo JJ (2011). Velocity loss as an indicator of neuromuscular fatigue during resistance training. Med Sci Sports Exerc.

[21] Rodríguez-Rosell D, Yáñez-García JM, Mora-Custodio R, Sánchez-Medina L, Ribas-Serna J, González-Badillo JJ (2021). Effect of velocity loss during squat training on neuromuscular performance. Scand J Med Sci Sports.

[22] Chan AW, Tetzlaff JM, Altman DG, Laupacis A, Gøtzsche PC, Krleža-Jerić K, Hróbjartsson A, Mann H, Dickersin K, Berlin JA, Doré CJ, Parulekar WR, Summerskill WS, Groves T, Schulz KF, Sox HC, Rockhold FW, Rennie D, Moher D (2013). SPIRIT 2013 statement: defining standard protocol items for clinical trials. Ann Intern Med.

[23] Greenwood SA, Koufaki P, Mercer TH, Rush R, O'Connor E, Tuffnell R, Lindup H, Haggis L, Dew T, Abdulnassir L, Nugent E, Goldsmith D, Macdougall IC (2015). Aerobic or resistance training and pulse wave velocity in kidney transplant recipients: a 12-week pilot randomized controlled trial (the Exercise in Renal Transplant [ExeRT] trial). Am J Kidney Dis.

[24] Ferguson TW, Komenda P, Tangri N (2015). Cystatin C as a biomarker for estimating glomerular filtration rate. Curr Opin Nephrol Hypertens.

[25] Levey AS, Stevens LA (2010). Estimating GFR using the CKD Epidemiology Collaboration (CKD-EPI) creatinine equation: more accurate GFR estimates, lower CKD prevalence estimates, and better risk predictions. Am J Kidney Dis.

[26] Ostrowski K, Rohde T, Asp S, Schjerling P, Pedersen BK (1999). Pro- and anti-inflammatory cytokine balance in strenuous exercise in humans. J Physiol.

[27] Lipsy RJ (2003). The National Cholesterol Education Program Adult Treatment Panel III guidelines. J Manag Care Pharm.

[28] Marfell-Jones M, Olds T, Stewart A, Carter L (2006). International Standards for Anthropometric Assessment.

[29] Ramírez-Vélez R, Peña-Ibagon JC, Martínez-Torres J, Tordecilla-Sanders A, Correa-Bautista JE, Lobelo F, García-Hermoso A (2017). Handgrip strength cutoff for cardiometabolic risk index among Colombian children and adolescents: the FUPRECOL Study. Sci Rep.

[30] BC-1500 InnerScan PRO segmental body composition scale. Tanita Corporation.

[31] Peña Ibagon JC, Pinto EM, Collazos Morales CA, Rojas-Valverde D, Cardozo LA, Pardo YR, Felipe Martin W, Camilo Peña C (2024). Muscle strength as a marker of metabolic health in kidney transplant recipients: a cross-sectional study. J Bodyw Mov Ther.

[32] Pacheco-Herrera JD, Ramírez-Vélez R, Correa-Bautista JE (2016). Índice general de fuerza y adiposidad como medida de la condición física relacionada con la salud en niños y adolescentes de Bogotá, Colombia: Estudio FUPRECOL [Article in Spanish]. Nutr Hosp.

[33] Martínez-Cava A, Hernández-Belmonte A, Courel-Ibáñez J, Morán-Navarro R, González-Badillo JJ, Pallarés JG (2020). Reliability of technologies to measure the barbell velocity: implications for monitoring resistance training. PLoS One.

[34] Arango Vélez EF, Echavarría Rodríguez AM, Aguilar González FA, Patiño Villada FA (2020). Validation of two questionnaires to assess the level of physical activity and sedentary time in a university community in Colombia [Article in Spanish]. Rev Fac Nac Salud Pública.

[35] Español-Moya MN, Ramírez-Vélez R (2014). Psychometric validation of the International FItness Scale (IFIS) in Colombian youth [Article in Spanish]. Rev Esp Salud Publica.

[36] Cohen J (1988). Statistical Power Analysis for the Behavioral Sciences Second Edition.

