# Supplementary material for: Effects of Velocity-Based Resistance Training on Renal Function and Metabolic Health in Kidney Transplant Recipients: Protocol for a Pilot Randomized Controlled Trial
Source: JMIR Res Protoc. 2026 Jul 10;15:e94010. doi: 10.2196/94010 (PMC13401074; doi:10.2196/94010)
Supplement: Multimedia Appendix 1 [file resprot_v15i1e94010_app1.docx]

**Multimedia Appendix 1. SPIRIT 2025 Checklist**

| **Section** | **Item** | **Description** | **Manuscript Location** | **Status** |
| --- | --- | --- | --- | --- |
| Administrative | Title | Identification as a protocol of a randomized trial | Title page | Completed |
| Administrative | Trial registration | Trial identifier and registry name | Abstract; Methods | Completed |
| Administrative | Funding | Sources and types of financial or material support | Funding section | Completed |
| Administrative | Roles and responsibilities | Author contributions and responsibilities | Authors’ Contributions | Completed |
| Introduction | Background and rationale | Scientific background and justification | Introduction | Completed |
| Introduction | Objectives | Specific objectives or hypotheses | Abstract; Introduction | Completed |
| Methods | Study design | Description of trial design (e.g., pilot RCT, allocation ratio) | Study Design section | Completed |
| Methods | Study setting | Description of study setting and location | Participants section | Completed |
| Methods | Eligibility criteria | Inclusion and exclusion criteria | Participants and eligibility criteria | Completed |
| Methods | Interventions | Detailed description of interventions for each group | Intervention section | Completed |
| Methods | Outcomes | Clearly defined primary and secondary outcomes | Outcome Measures | Completed |
| Methods | Outcome hierarchy | Predefined prioritization of outcomes | Outcome Measures | Completed |
| Methods | Participant timeline | Schedule of enrolment, interventions, and assessments | Instruments and Procedures; Intervention | Completed |
| Methods | Sample size | Justification of sample size | Sample Size section | Completed |
| Methods | Sequence generation | Method used to generate random allocation sequence | Randomization section | Completed |
| Methods | Allocation concealment | Mechanism to conceal allocation sequence | Randomization section | Completed |
| Methods | Implementation | Who generates sequence and assigns participants | Randomization section | Completed |
| Methods | Blinding | Who is blinded and how | Blinding section | Completed |
| Methods | Data collection methods | Methods for data collection and measurement | Instruments and Procedures | Completed |
| Methods | Data management | Data entry, coding, storage, and quality control | Data Management section | Completed |
| Methods | Confidentiality | Measures to protect participant confidentiality | Ethical Considerations | Completed |
| Methods | Statistical methods | Statistical analysis plan for outcomes | Statistical Analysis | Completed |
| Methods | Missing data | Handling of missing data | Statistical Analysis | Completed |
| Methods | Additional analyses | Planned additional/sensitivity analyses | Statistical Analysis | Completed |
| Monitoring | Data monitoring | Data monitoring procedures or justification for absence | Safety Monitoring | Completed |
| Monitoring | Harms | Monitoring and reporting of adverse events | Safety Monitoring | Completed |
| Monitoring | Auditing | Auditing procedures | Not applicable (pilot trial) | Acceptable |
| Ethics | Ethics approval | Ethics committee approval and reference number | Ethical Considerations; Results | Completed |
| Ethics | Consent | Informed consent procedures | Ethical Considerations | Completed |
| Ethics | Dissemination policy | Plans for dissemination of results | Discussion | Completed |
| Results | Trial status | Recruitment status and study progress | Results section | Completed |
| Results | Timeline | Dates of study phases and expected completion | Results section | Completed |
